# The use of virtual reality simulation to facilitate surgical ward-based learning in medical students during the COVID-19 Pandemic

**DOI:** 10.12669/pjms.37.2.4118

**Published:** 2021

**Affiliations:** 1 Isabelle Carty, MBBS. Medical Education Fellow, Dartford, UK; 2 Rupali Shah, MBBCh. Surgical Simulation Fellow, London, UK

The use of virtual reality simulation is vital especially in this current climate in order to immerse students in a medical environment and to improve their confidence in dealing with patients and other healthcare professionals especially when they are deprived of hands on experience on the wards.^1^-^4^

The unprecedented Covid-19 pandemic of 2020 quickly resulted in the removal of all non-essential personnel from all wards and emergency departments.^5^ This had the unfortunate result of suspending medical student placements which are heavily relied upon to provide the students with necessary “hands-on” experience.^5^,^6^ In the absence of this resource, an alternate method involving the use of Virtual Reality (VR) simulations was employed in order to ensure that the students had a comparable experience.

VR simulations were used to simulate the ward / emergency room environments and the students were able to gain valuable experience with numerous common scenarios that they might encounter on the surgical wards. Practically, there were two students in the simulation room at a time, two metres apart and both wearing surgical masks while the VR hardware employed disposable sanitary covers which were changed when the hardware was wiped down with antibacterial wipes after each use. For each VR session, each student was allocated a 1-hour time slot thereby ensuring that there was enough time for each student to experience up to three simulations with a debriefing session after each.

The digital immersive environment allowed students to build upon their didactic preparation and previously acquired knowledge base. In the simulations, they were able to take patient histories and examine the patients as well as simulate tasks like taking bloods, reading an ECG, prescribing medication and asking the simulated nurse to put up fluids or catheterise the patient.

Feedback was given at the end of each simulation in the form of a debriefing session run by the simulation facilitator. Using the technical proficiency that they had acquired in the first scenario, the students went on to employ these skills directly in their next scenarios thereby capitalising on the repetition of core skills to maximize their learning.

As the world becomes increasingly technologically advanced, simulation experiences in the healthcare and medical education paradigm are now evolving to include digital experiences as well. In this instance, VR was successfully used to enhance both knowledge acquisition and experience. Feedback from the participating students was overwhelmingly positive with many suggesting that they were more comfortable learning in a simulated environment prior to being on the wards and they found that this led to increased levels of confidence in their skills suggesting that even in the absence of a pandemic, there is a place for VR simulation in medical education.
